# Differential Immune Response Following Intranasal and Intradermal Infection with *Francisella tularensis:* Implications for Vaccine Development

**DOI:** 10.3390/microorganisms9050973

**Published:** 2021-04-30

**Authors:** McKayla J. Nicol, David R. Williamson, David E. Place, Girish S. Kirimanjeswara

**Affiliations:** 1 Department of Veterinary and Biomedical Sciences, The Pennsylvania State University, University Park, PA 16802, USA; mjn5181@psu.edu (M.J.N.); dave.williamson10@gmail.com (D.R.W.); David.Place@stjude.org (D.E.P.); 2 Pathobiology Graduate Program, The Pennsylvania State University, University Park, PA 16802, USA; 3 Clinical and Translational Sciences Graduate Program, The Pennsylvania State University, University Park, PA 16802, USA; 4 The Center for Molecular Immunology and Infectious Disease, The Pennsylvania State University, University Park, PA 16802, USA; 5 The Huck Institutes of the Life Sciences, The Pennsylvania State University, University Park, PA 16802, USA

**Keywords:** *Francisella tularensis*, disparate routes of infection, vaccine development, immune response

## Abstract

*Francisella tularensis* (*Ft)* is a Gram-negative, facultative intracellular coccobacillus that is the etiological agent of tularemia. Interestingly, the disease tularemia has variable clinical presentations that are dependent upon the route of infection with *Ft*. Two of the most likely routes of *Ft* infection include intranasal and intradermal, which result in pneumonic and ulceroglandular tularemia, respectively. While there are several differences between these two forms of tularemia, the most notable disparity is between mortality rates: the mortality rate following pneumonic tularemia is over ten times that of the ulceroglandular disease. Understanding the differences between intradermal and intranasal *Ft* infections is important not only for clinical diagnoses and treatment but also for the development of a safe and effective vaccine. However, the immune correlates of protection against *Ft*, especially within the context of infection by disparate routes, are not yet fully understood. Recent advances in different animal models have revealed new insights in the complex interplay of innate and adaptive immune responses, indicating dissimilar patterns in both responses following infection with *Ft* via different routes. Further investigation of these differences will be crucial to predicting disease outcomes and inducing protective immunity via vaccination or natural infection.

## 1. Introduction

Because of its highly infectious nature and relatively high mortality rate in untreated individuals exposed to aerosolized bacteria, much of the immunology of the host-pathogen interactions with *Francisella tularensis* (*Ft)* has been studied in the context of the respiratory tract. Intranasal infections result in pneumonic tularemia, which is well-known as the most acute form of the disease and is generally characterized by fevers, flu-like symptoms, and difficulty breathing. While this is the most likely route of exposure for weaponized bacteria, *Ft* also has the ability to infect through the gastrointestinal tract by ingestion and through the skin via handling of infected animals or bites of infected arthropods or insects [1]. These alternative routes of infection result in one of the other two main clinical presentations of tularemia, which are oropharyngeal and ulceroglandular, respectively. These forms of the disease are characterized by skin ulcers or stomach ulcers, swollen lymph nodes, and fever [2].

Worldwide incidence rates of tularemia are not available as they are not reported to the World Health Organization. However, sufficient local data indicate that ulceroglandular tularemia is the most common form of the disease [3]. Furthermore, most naturally occurring infections are due to arthropod bites or ingestion of contaminated water, resulting in mainly self-limiting disease associated with low mortality rates [4]. Specifically, mortality rates for ulceroglandular and oropharyngeal tularemia are under 5% [5]. In contrast, pneumonic tularemia has a mortality rate of up to 60% without therapeutic intervention, which is a major contributing factor to the classification of this pathogen as a Tier 1 Select Agent [6].

Differences between intradermal and intranasal infections with *Ft* are important not only for clinical diagnoses and treatment but also for the development of a safe and effective vaccine. The vaccine candidate that has been studied best to date, *F. holarctica* Live Vaccine Strain (LVS), was developed over 50 years ago [7]. The basis of attenuation for this strain is still not well defined, and its potential for reversion is concerning [7]. Furthermore, the protection offered by LVS is dependent upon the delivery method, with intranasal delivery being most effective, but it also has significant potential adverse effects [8]. Relative to other intracellular pathogens, the immune correlates of protection against *Ft* have been somewhat poorly described. Additionally, it can have different outcomes in different strains of mice because the bacteria can be introduced through multiple different routes. Moreover, multiple strains of *Ft* exist, leading to many conflicting claims related to the immune mediators for protection [9]. Newer strategies for manipulating the development of protective immune responses include prime-boost vaccines, which may target different sites with different vaccine components, and adjuvants to increase protection [10,11]. Additionally, a clearer understanding of the differences in the basic immune response to *Francisella* in the skin vs the respiratory tract may be required to understand why scarification or intramuscular vaccines fail to elicit efficient protection against intranasal challenges with this pathogen. This review will examine what is known regarding the various models used to study intradermal vs intranasal infections with *F. tularensis*. We will also provide an analysis of overall differential immune responses and their key impacts on vaccine development.

## 2. Vector-Borne Intradermal Infections and Aerogenic Exposure

There are multiple vectors capable of carrying *Ft* and infecting humans through the intradermal route. Tabanids such as horse and deer flies transmit *Ft* to humans through mechanical biting and seek multiple hosts as a part of their feeding strategy, which leads to multiple exposures with the potential for local outbreaks of tularemia [12,13,14]. Mosquitoes have also been shown to transmit *Ft* to many small mammals, but they are poor long-term hosts for the bacterium [1,15]. Finally, tick-borne *Ft* has been linked to several outbreaks, and the bacteria has been shown to replicate well within these hosts, suggesting that they are a major cause of transmission in nature [16,17,18,19].

There is much less data on outbreaks of naturally occurring primary pneumonic tularemia, acquired via inhalation of *Ft*, as these cases are much less common. However, over the years, a few cases of unintentional aerosolization of the bacteria by different means have been recorded. One of the larger unintentional outbreaks of primary pneumonic tularemia in the US has been attributed to mechanical aerosolization of *Ft* via lawn mowing in Martha’s Vineyard. It has been hypothesized that urine and feces from infected rodents were shed into the grass and subsequently aerosolized by lawn mowers [20]. A similar case occurred in Europe with farmers and contaminated hay [21]. Most other recorded cases are attributed to disturbing infected rabbit carcasses or transmission through household pet hair and dander that were previously in contact with infected rabbits [22,23,24,25].

## 3. Intradermal and Intranasal Infection Models for LVS

Numerous species have been used to study the various aspects of immunity to different *Ft* strains and vaccine candidates, including rabbits, multiple strains of laboratory mice, rats, guinea pigs, rhesus macaques, African green monkeys, cynomolgus monkeys, and even humans [26,27,28,29,30,31,32,33]. For most animal models, however, very basic characteristics of the immune responses have been measured, and studies have largely focused on the host’s susceptibility to different substrains of *Ft*. This section will discuss overviews of these different models, highlighting what has been observed regarding intranasal vs intradermal infection and vaccination responses.

### 3.1. Rabbits

#### 3.1.1. Rabbits–Intradermal

In cottontail rabbits (*Sylvilagus spp.)*, comparisons between intradermal infections with the more virulent type A (SchuS4) and less virulent type B (LVS) strains of *Ft* were performed, and it was shown that type A strains were highly pathogenic, killing animals between days 3–13 and causing micro-abscesses in livers and spleens, whereas animals infected with type B strains, which were rarely lethal, only experienced mild fever and lethargy with antibody responses peaking between day 14–21 [31]. While these outcomes are similar to those seen from infections in mice, they differ somewhat from what is observed in humans, where the disease is more severe with type A1b, followed by A1a and type B, but not type A2 strains [34,35,36]. Rabbits have also been used to study attenuated SchuS4 strains by scarification (SchuS4 Δ*guaBA*, Δ*aroD*, and Δ*fipB* strains) and LVS with Δ*guaBA* and Δ*aroD* mutants protecting 27%–36% of rabbits and prolonging time to death, but LVS was unable to fully protect rabbits from a secondary aerosolized SchuS4 challenge [36]. Additionally, the only local immune responses described in the skin show that injection of LVS at high doses leads to erythema and edema locally and that scarification, compared to intradermal and subcutaneous injection, leads to higher titers of *Ft*-specific IgA [37].

#### 3.1.2. Rabbits–Intranasal

Rabbits have been shown to survive high dose LVS respiratory vaccinations, which provided significantly better protection and prolonged the time to death against the aerosolized SchuS4 challenge in comparison to scarification vaccines [38]. Immune responses studied in response to intranasal LVS exposure in rabbits has been less extensive but does include changes in erythrocyte sedimentation rates (ESR), complete blood counts, and IgG titers against *Ft*. It was shown that ESR and complete blood counts were not altered, but that IgG titers were significantly increased and corresponded to survival following the respiratory challenge with SchuS4 [36,38,39].

### 3.2. Rodents

The most common animal model for studying *Francisella* immunology is the mouse (*Mus musculus*). The power of this animal model lies in the wide availability of gene knockouts of the immune system, the readily available immunological tools, and the susceptibility of mice to even low doses of multiple strains of *Ft.* All of these elements are extremely useful for determining essential immune functions. Much of the focus to date in mouse model studies has been on how the different routes of vaccination differ in their levels of protection against intranasal challenges and the impact that the route of vaccination has on the subsequent immune response in the respiratory and systemic organs. However, the local immune response in the skin, which may have profound implications for improving future intradermal vaccines, requires further study.

Furthermore, although mouse models are widely used, one of the major limitations that has been identified for using mice to study immune responses to *Ft* is that mice have an increased sensitivity to strains that cause mild or non-existent symptoms in humans. Therefore, rat (*Rattus)* models (F344) were introduced, as their susceptibility to primary infection by different strains has been demonstrated to be more similar to that of a human [40]. Finally, guinea pigs (*Cavia porcellus*) have also been used, specifically to study tick transmission as well as the pathogenesis of tularemia [41].

#### 3.2.1. Rodents–Intradermal

The two main strains of mice that are used to study *Ft* are BALB/c and C57BL/6 mice. While some differences exist, many features of the immune response to infection with *Ft* are shared between the two mouse strains, including the protective roles of IFNγ, TNFα, and α/β T cells [42,43,44,45,46]. While much is known about the immune response to LVS and type A strains in the respiratory tract and systemic organs of infected mice, relatively little is understood about the local immune response in the skin of mice following intradermal vaccination and/or challenge with a vaccine or virulent strains. Only a handful of studies have looked at the local skin immune response, but what has been shown is that both of the strains of mice upregulate IL-1β, IL-6, IL-12p35, MIP-2 (CXCL2), KC (CXCL1), MCP-1 (CCL2), IP-10 (CXCL10), MIP-1α (CCL3), MIP-1β (CCL4), and RANTES (CCL5). BALB/c but not C57BL/6 upregulate IFNγ and IL-10. Additionally, C57BL/6 but not BALB/c upregulate IL-12p40, TNFα, and iNOS [47]. Another study has shown that granulocyte-macrophage colony-stimulating factor (GM-CSF) deficient C57BL/6 mice are more susceptible to intradermal infections with LVS than mice with normal GM-CSF levels [48]. It was also demonstrated that these mice had increased antibody production and T cell responses [48].

Yet another study has identified that BALB/c mice intradermally vaccinated with LVS express IL-12, TNFα, and IFNγ in the skin following a challenge with a lethal dose of LVS 5–6 weeks later. Further, the vaccinated mice prevented dissemination of the challenge LVS to livers and spleens [30]. Interestingly, BALB/c and C57BL/6 mice vaccinated intradermally results in different levels of protection against virulent *F. tularensis.* BALB/c strains are protected against intradermal challenge with both type A and B strains, but C57BL/6 mice are only protected against type B strains [49]. Additionally, naïve C57BL/6J mice are unable to survive intradermal infection by *F. tularensis* ssp. *novicida* and *F. tularensis* ssp. *tularensis* (SchuS4), whereas IFNAR1^-/-^ mice survive at a similar dose [50]. This observation has also been made with several other intracellular bacterial infections, including *Listeria monocytogenes, Chlamydia murinarum,* and *Mycobacterium tuberculosis* [51,52,53,54,55,56]. Additional mouse studies have shown that neutrophils have also been largely implicated in protection against primary intradermal LVS infection [57]. It has also been suggested that different phagocytic cells disseminate LVS from the skin than their respective counterparts in the respiratory tract. Specifically, interstitial macrophages and neutrophils containing LVS in the lungs are detected following intradermal infection. However, the specific cell tropism in the skin following intradermal infection has not been as well characterized [58].

The introduction of rat models has provided some insight into immune responses to primary infections. However, rats have been almost exclusively studied in the context of intranasal or aerosolized infections and vaccinations [40,59,60], except for one study, which showed that aerosol, intranasal, intraperitoneal, intramuscular, and subcutaneous vaccination methods were all equally protective against a secondary challenge with SchuS4 [41]. This lack of variation in the protection offered through alternative locations of vaccine delivery may suggest, however, that the F344 rat model for *Ft* infection may not be very closely related to the human response, particularly for vaccine studies.

Finally, guinea pig models have largely been used to examine the capability of ticks to act as reservoirs for *Ft.* However, there have been additional studies investigating guinea pigs’ immune response not only during infection but also following subcutaneous injection with rabbit immune serum against *Ft* followed by a challenge [61]. Additionally, the sensitivity of guinea pigs to the different strains of *Ft* has been studied, and it has been shown that guinea pigs do have similar sensitivity to mice in regards to the different strains [41]. However, a much deeper understanding of immune responses in this model is lacking.

#### 3.2.2. Rodents–Intranasal

Several of the features identified to be important for the murine immune response to intradermal infection with *Ft* are also essential during intranasal infections. Specifically, IFNγ, TNFα, and α/β T cells all play an important role [42,43,44,45,46]. It has also been established that the initial macrophage population infected in the lungs when LVS is given intranasally in the alveolar macrophages rather than the interstitial population seen in intradermal infections [58]. Furthermore, vaccination of BALB/c and C57BL/6 mice with LVS intradermally leads to different levels of protection against intranasal secondary challenge. Specifically, BALB/c are protected against type B aerosol challenges, whereas C57BL/6 are not. Furthermore, neither strain of mice is protected against aerosol challenge with type A [49]. Another major difference that has been identified between the two strains of mice during intranasal infections that may contribute to these phenotypes is that C57BL/6 mice have a T cell response that is skewed towards Th1 populations, while the response in BALB/c mice is predominantly Th2 regulated [62]. It also follows that the macrophage populations are likely skewed to M1 and M2 macrophage phenotypes for C57BL/6 and BALB/c mice, respectively, during infection [63]. Furthermore, GM-CSF deficient mice are more resistant to intranasal infection with both type A and B strains of *Ft* [48].

### 3.3. Non-Human Primates

#### 3.3.1. Non-Human Primates–Intradermal

Non-human primates have also been used to study immunity to *Ft,* including rhesus monkeys (*Macaca mulatta*), African green monkeys (*Chlorocebus aethiops*), marmosets (*Callithrix jacchus*) and cynomolgus monkeys (*Macaca fascicularis*) [28,64,65,66]. However, the dermal immune response has been poorly characterized beyond local replication of LVS in the skin and local lymph nodes and the development of anti-*Francisella* antibodies. Similar to mouse models, dermal vaccination provided less protection than respiratory vaccination with LVS in monkeys and did not delay enlargement of trachea-bronchiolar lymph nodes. However, these vaccinations were able to prevent fatal septicemia, which developed in all unvaccinated animals when challenged with virulent SchuS4 [65].

#### 3.3.2. Non-Human Primates–Intranasal

Each of the previously described primate models was susceptible to lethal respiratory infection by virulent *Ft.* However, respiratory LVS vaccination was able to protect against aerosol challenge with type A strains, which is similar to what has been demonstrated in humans [8]. In fact, primate models of *Ft* are thought to most closely replicate human disease in comparison to all other animal models investigated thus far [67]. It was also determined that anti-*Francisella* antibodies were significantly higher in respiratory vaccinated animals as compared with intradermally vaccinated animals [65].

### 3.4. Humans

#### 3.4.1. Humans–Intradermal 

Despite LVS being an experimental vaccine and virulent *Ft* having a high mortality rate, human subjects were used in vaccination and challenge studies in the past. Prior to the development of the LVS strain, the Foshay killed-bacteria vaccine was tested and shown to elicit weak antibody responses. This vaccine failed to protect against local skin lesions in intradermally challenged individuals [68,69]. In unvaccinated individuals, intracutaneous inoculation lead to local skin lesions, antibody responses, regional adenopathy, and the majority of individuals required antibiotic treatment due to systemic effects including fever, extreme weight loss, and fatigue [33]. A second study of subjects vaccinated with the Foshay vaccine and challenged cutaneously resulted in the majority of the subjects experiencing local lesions, while a minority showed systemic disease requiring treatment by antibiotics. Yet another subset of subjects required removal or drainage of lymph nodes months after the secondary challenge [33]. The booster vaccination with the Foshay vaccine 6–8 months after initial vaccination also failed to protect from local lesions, adenopathy, and systemic disease [33]. In the same study, individuals who were challenged with virulent *Ft* and treated with antibiotics were re-challenged and showed no protection to skin lesions and mild protection of systemic disease in only a few individuals [33].

More recently, the LVS vaccine testing in humans was performed by scarification and subcutaneous inoculation, which were well tolerated at all doses with increasing doses correlating to higher serum IgG, IgM, IgA, and IFNγ responses [70]. While antibodies are useful indicators for exposure to *Francisella*, they are not yet correlated with full protection in humans [71]. These studies also revealed that the LVS bacteria were not detected in any blood samples, suggesting it is restricted mainly to the skin in humans following scarification or subcutaneous vaccination [70]. Furthermore, while microagglutination titers in this study correlated with the degree of reddening of the skin, IFNγ correlated with hardening of the injection site [70]. Considering the vast majority of LVS appears to remain in the skin of vaccinated humans, understanding how the immune system is interacting with the infection could reveal ways to generate greater protection during intranasal or respiratory infections.

#### 3.4.2. Humans–Intranasal

Initial studies comparing the scarification vaccines of the LVS strain to Foshay-killed bacteria in humans showed that LVS increased protection from disease following low-dose aerosol challenge, but the protection was overcome by increasing challenge doses [32,72]. While high-dose aerosol vaccination with LVS was shown to be more protective than low-dose aerosol or scarification methods, the logistics and safety concerns of aerosolized vaccinations led to scarification being adopted in the US as the main vaccination method [8].

### 3.5. Animal Model Overview

Overall, a great deal of information has been obtained from the various animal models discussed, and each has made its own unique contributions towards our understanding of the differences in host immune responses to *Ft* during infection by disparate routes. Although none of the animal models exactly replicate the human responses to each *Ft* strain, based on the collective data, we can identify advantages and disadvantages of intranasal and intradermal animal models of infection and, more importantly, vaccination. Specifically, intradermal vaccinations can induce systemic responses in any of the above-mentioned models. However, mucosal-specific protective immune responses are severely lacking in intradermal vaccinations in comparison to those given by an intranasal route in most cases. Additionally, despite the fact that almost all of the tested models showed improved protection with a respiratory vaccination, they were largely unable to provide full protection against an aerosol challenge with type A, virulent *Ft* strains. Therefore, further studies must be completed and confirmed by multiple models in order to identify the necessary correlates of the immune response that will provide such protection.

## 4. Immune Response and Vaccines

As discussed above with all of the different models, while there are multiple vectors which transmit the bacteria intradermally, how the local immune response in the skin of these hosts interacts with the bacteria and directs the adaptive immune response is not well understood. Furthermore, few correlates of the immune system are known to be protective against challenge with highly virulent *Ft*, and the reason for differences in protective immunity are also not well understood. In this portion of the review, we will focus on what is known regarding the immunology associated with infection by LVS and the more virulent type A strains of *Ft.* More specifically, we will focus on how different T cells develop during primary infection and provide memory responses to secondary challenge, as it is very possible that differences in the overall T cell response during intranasal vs intradermal infections are paramount in the disparate mortality rates between the two infection routes.

### 4.1. Primary Infection

**Figure 1:**
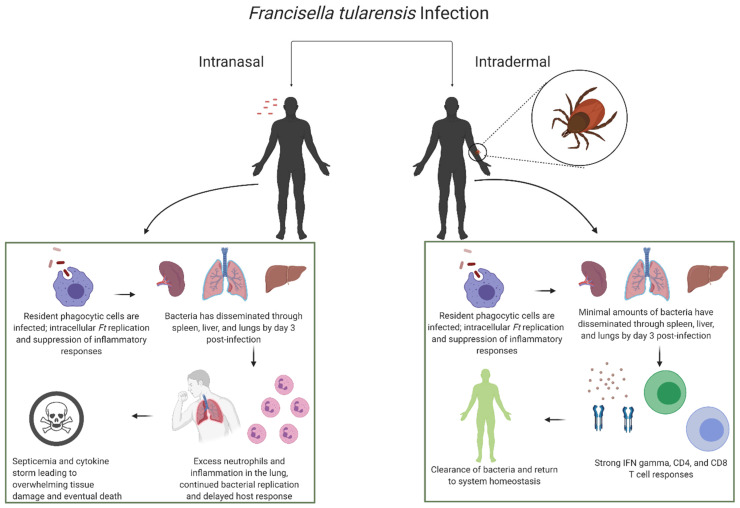
General characteristics of the host’s immune response during an untreated intranasal or intradermal infection with *Francisella tularensis.* Created with BioRender.com.

Both humoral and cell-mediated immunity during infection with *Ft* have been investigated to an extent for both IN and ID infections, and it is generally accepted that synergistic effects between these two aspects of host immunity are necessary for the successful clearance of the pathogen (Figure 1). Regarding the cellular milieu, tissue-resident macrophages, including alveolar macrophages in the lung and Langerhans cells in the skin, make up the majority of the initially infected cell populations during IN and ID infections, respectively. However, major differences, including an increased percentage of infected neutrophils and interstitial rather than alveolar macrophages in the lung during the initial stages of ID infection, likely contribute to the differences in mortality between the two routes of infection [58]. Once the bacteria have been taken up by the tissue-resident macrophages and other phagocytic cells, they are able to escape into the cytosol, replicate, leave the cell, and disseminate throughout the host. During this initial phase, the host inflammatory responses are minimal due to suppressive mechanisms from *Ft*; however, once the bacteria have begun to disseminate throughout the host, increased cytokine expression and inflammatory markers have been identified. Specifically, IFNγ, TNFα, IL-6, IL-17, and IL-12 all have been identified as critical factors for the clearance of the bacteria. In fact, the most well-characterized cytokine involved in protection from *Francisella* is IFNγ, which activates phagocytic cells and increases the production of reactive oxygen species (ROS) in order to limit replication of *Ft* [73,74]. LVS, as an attenuated strain, is more susceptible to killing by ROS than SchuS4, which possesses enzymes that neutralize ROS [73]. Initially, IFNγ is primarily produced by natural killer (NK) cells, which are recruited by 72hr post-inoculation [75]. Additional immune mediators such as TNFα, reactive nitrogen intermediates, TLR2, and the inflammasome have been identified following infection with *Ft* [74]. The role of type I interferons, such as IFNβ, remains to be fully elucidated, as they have been implicated in both inhibiting gamma delta T cell production of IL-17A and activating the inflammasome during infection [50,76]. Though IL-17 protects against primary infection with LVS, it does not appear to be required for protection against LVS secondary challenge or primary challenge with virulent *Ft* [77]. Interestingly, the majority of the critical cytokines identified to play a role during infection with *Ft* also appear to be differentially regulated based on the route of infection [73,74,78,79,80,81,82,83]. Finally, it has been shown that serum antibody levels, although associated with the infectious dose, are not necessarily correlated with protection in humans [84]. Transfer of serum from previously infected mice was unable to protect mice with suppressed T cell responses, although it was able to offer some protection to WT mice [71,85,86]. These data indicate that host antibody responses may be necessary but not sufficient to offer protection from *Ft* infection. Furthermore, it has been established that either CD4+ or CD8+ T cells are required for protection against *Ft* [78,87,88]. This has been demonstrated through significantly increased survival rates as a result of the transfer of naïve splenic lymphocytes depleted of B220+ cells to immunocompromised mice and the inability of athymic T-cell deficient *nu/nu* mice to survive infection [89]. Interestingly, there have also been differences detected in the expansion of T cell populations between IN and ID infections [90].

### 4.2. Vaccination

Many intracellular pathogens require cell-mediated immunity to kill infected host cells. T cells are associated with protection to challenges with virulent *Ft* through their ability to prevent dissemination of bacteria from the respiratory tract, though this is still poorly understood [79]. Furthermore, it is possible that the differences in immune response between intranasal and intradermal infections with *Ft* are due to disparate recruitment and retention of memory T cells as well as their ability to mount appropriate effector responses. CD8^+^ T cells are particularly well-suited to recognize cells infected with intracellular pathogens like *Ft* via intracellular peptides presented by MHC class I (MHC-I). The contribution of CD8^+^ T cells to protective immunity against *Ft* is well recognized, and the number of effector CD8^+^ T cells in the lungs and spleen correlates with protection from re-challenge [91,92]. Furthermore, it has been determined that whole-splenocyte or CD8-enriched preparations from vaccinated animals can limit the intracellular proliferation of LVS in murine bone-marrow-derived macrophages(BMDMs) [83,93,94,95], and the ability to do so correlates with the protective efficacy of the vaccine strain [95]. However, whole-splenocytes fail to control the intracellular growth of the virulent SchuS4 strain in the same assay unless they are pre-activated with PMA/ionomycin [92]. Additionally, past studies in which whole-splenocyte preparations from animals vaccinated with more protective sub-strains of LVS show superior control of intra-macrophage bacterial growth ex vivo [92,94].

The depletion of CD8^+^ T cells from vaccinated animals eliminates the otherwise protective effects of vaccination [78,96], and the transfer of T cells to immunocompromised (scid) mice allows for survival of otherwise lethal *Ft* challenge [89]. In contrast, the transfer of immune serum fails to protect recipients from virulent *Ft* [71,85,86], implying that antibodies are not sufficient for protective immunity. While it is clear that CD8^+^ T cells contribute to protective immunity against *Ft*, how they do so is not well understood, and protection varies based on the route of exposure.

It has been well established that naïve CD8^+^ T cells circulate in the blood and secondary lymphoid organs where they interact with professional antigen-presenting cells (APCs) and respond to cognate peptides. Upon activation, CD8^+^ T cells undergo rapid expansion and subsequently home to sites of infection. These cells acquire effector functions that allow them to kill infected cells, such as those invaded by *Ft,* via the release of perforin and granzymes into the target cell [97,98]. Additionally, CD8^+^ T cells can induce apoptosis through binding of the Fas ligand when the Fas receptor is present on the target cell [99]. In addition to their direct cytotoxic actions, effector CD8^+^ T cells also produce a number of inflammatory cytokines that help control infection and stimulate other arms of the immune response. The most-studied subset of CD8^+^ T cells produces IFN-γ and TNF-α; however, a number of less common, more recently characterized subsets produce a more diverse array of cytokines, including IL-4, IL-10, IL-17, and TGF-β, several of which are crucial to the clearance of *Ft* [100]. Following the clearance of infection, there is a contraction of the T cell response, as 90–95% of pathogen-specific T cells undergo apoptosis [97]. Nonetheless, a substantial pool of memory cells with functionally superior abilities to respond to re-infection are left behind [97]. These memory cells include subsets of central memory (T_CM_), effector memory (T_EM_), and resident memory (T_RM_), each of which plays a distinct role during a memory immune response [101,102,103]. The relative importance of each of these memory subsets for effective protection against secondary infection with *Ft* remains to be established.

An interesting subset of CD4^−^CD8^−^ double-negative T cells have been shown to produce IL-17A and IFNγ in response to intranasal LVS in the lungs, but whether they are involved in memory has not been evaluated [104]. While it is known that CD4+, CD8+, and CD4^−^CD8^−^ double-negative T cell responses arise during vaccination with LVS, their localization and effector functions remain poorly characterized, and whether they are essential for protection is uncertain [42,88,104,105]. Furthermore, the differences in protection to challenge are highly dependent upon the route of vaccination (intranasal or intradermal), suggesting that T cells are endowed with different properties by these two different routes. It has been suggested that differences in the initial phagocytic cell subsets infected by intradermal and pulmonary infection lead to differences in the T cell responses during the challenge, given that they may have differences in their antigen presentation capabilities, activation, or localization [58,90]. However, to date, studying the pathogen-specific cells that arise during vaccination has been hampered by the lack of tools to study them. The only *Francisella*-specific epitope described is the glycoprotein Tul4_86-99_ epitope (RLQWQAPEGSKCHD), and an MHC-II tetramer has been generated for this epitope by the NIH Tetramer Facility but has not been shown to stain antigen-specific cells [106,107]. A recent development on this front was the generation of an ovalbumin-expressing strain of LVS that enabled the identification of both temporal and spatial characteristics of various subsets of T cells [84]. However, the relative roles of those antigen-specific T cells still remain to be studied.

The memory responses elicited by an infection (or vaccine) depend on multiple factors, including the intensity and duration of antigen exposure [108], the inflammatory milieu [109], and the route of exposure [103,108,110]. For *Ft* infections, in order to develop resident memory cells in the respiratory tract, it appears that priming by the respiratory route is required. A similar phenomenon was found following the vaccination of mice to influenza, where a respiratory delivery leads to the formation of lung T_RM_, while intraperitoneal delivery of the vaccine does not elicit such a response [111]. In humans, the lungs have an enrichment of CD8^+^ T_RM_ targeting respiratory pathogens, such as influenza and respiratory syncytial virus, but not following an infection with blood-borne pathogens, such as the cytomegalovirus and Epstein–Barr virus [112]. It is conceivable that the formation of lung T_RM_ contributes to the superior protection elicited by inhalational tularemia vaccines. Consistent with this, aerosol LVS-vaccinated mice have lymphoid aggregates in perivascular and peribronchial areas of the lung, while mice receiving an intradermal vaccine do not [78]. Besides the site-specific generation of T_RM_, it is likely that the route of infection/vaccination will affect the immune response in additional ways. It has been reported that intranasal vs intravenous inoculation leads to a different ratio of T_CM_ to T_EM_ [110]. The distribution of different classes of antigen-presenting cells is one potential explanation; certain classes have been shown to favor either T_EM_ or T_CM_ differentiation [113]. An additional, though not exclusive, explanation involves the inflammatory cytokine profile induced in each tissue, which is known to affect both effector subsets [100] and memory development [109]. Collectively, our overall poor understanding of the dermal immune response is especially troubling given that vaccination with LVS is primarily administered by scarification of the skin, which is more likely to be used than intranasal vaccination [16,26,32,33,72,91]. However, as discussed above, it has been known for some time that LVS vaccinations can elicit robust protection against virulent strains, but only when given as a respiratory vaccine. Vaccination via the respiratory route, however, is associated with significant adverse effects, in contrast to the skin scarification route, which is safer but confers inferior protection in multiple infection models, including humans, as shown in Table 1.

**Table 1:** A summary of the data from studies comparing vaccination routes and protection elicited by LVS. All data shown are for respiratory challenges with virulent subsp. *F.* *tularensis* strains. “Protection” is defined as no need for antibiotic treatment for humans and survival for all others.

Species	% Protection (Respiratory Route)	% Protection (Non-Respiratory Route)	Non-Respiratory Vaccine Route	Refs.
Human	100	54	Scarification	[8]
Rhesus macaque	88	63	Intradermal	[61]
Guinea pig	60	5	Subcutaneous	[61]
Mouse (BALB/c)	40	0	Subcutaneous	[96]
Mouse (BALB/c)	60	0	Intradermal	[78]
Mouse (C57BL/6)	100 *	n.d.	n.a.	[92]
Mouse (C57BL/6)	0–10 *	0–10	Scarification, Intradermal, subcutaneous	[49,78,96,114]

* Protection of C57BL/6 mice is dependent on the substrain of LVS, ATCC. LVS fails to elicit protection, while RML LVS can provide complete protection. n.d. = not determined. n.a. = not applicable.

## 5. Conclusions

A fundamental prerequisite for the development of a vaccine that will be both safe and effective is the clear definition of the correlates of protective adaptive immunity during both primary and secondary infection with *Ft*. This information will lay the foundation for the design and evaluation of a vaccine that elicits protective immunity, mimicking that of respiratory LVS but with a superior safety profile. Novel adjuvants, for instance, could be developed to stimulate specific memory T cell responses without the need to give a live vaccine via the respiratory route, thus, decreasing potential adverse effects. Furthermore, the increasing importance of differences in the route by which vaccinations are given and their effect on challenge at distal mucosal sites has been appreciated in multiple pathogen models, which also lack successful vaccines today, including chlamydia, HIV, tuberculosis, and others [115,116,117,118,119,120]. Thus, the scope of this work increases to broader applications. Future work studying the differences between the intradermal and intranasal routes of vaccination and which components of the immune response are correlated with protection can lead to the development of better vaccines against the highly virulent *Ft* strains and will hopefully be translatable to vaccine development against the previously mentioned similar pathogens. Once important differences between routes of vaccination have been established and the immune response components have been clearly identified, studies can focus on generating a vaccine that elicits protection like that derived from intranasal LVS but without the safety concerns for adverse effects.
